# Macon Medical Society

**Published:** 1894-07

**Authors:** K. P. Moore

**Affiliations:** Reporter; Macon, Ga.


### *SOCIETY REPORTS.*

### MACON MEDICAL SOCIETY.

MACON, GA., March 20, 1894.

*President A. C. Blain, Presiding.*

It being the evening for report from the Section on Surgery, DR. E. G. FERGUSON, its Chairman, presented a case illustrating the good results from the use of Sayre's plaster jacket in spinal curvatures, especially when applied early in the disease. This patient was a boy six years old. He had been ill about two months when he came to the doctor for treatment. Patient had suffered great pain, general health greatly impaired, emaciation considerable, and the general outlook for permanent deformity and dwarfing, and possibly death, was very gloomy. The doctor put on the usual plaster jacket of Sayre, but patient was unable to wear it with any degree of comfort, and it was kept on but a few days. Another jacket was applied, which was worn with more comfort and for a much longer time. When this one became intolerable he made a new jacket, and as the disease yielded, and the marked deformity which existed at first gradually gave way, another jacket was adjusted, until now the little patient was wearing his fourth jacket with *perfect* comfort, and was rapidly regaining his health and flesh, and it was the opinion of the doctor that he would not only be entirely cured of his spinal disease, but that the deformity would be corrected. Dr. Ferguson thinks it is better to make a new jacket occasionally, and not to persist in the use of *one* while there is the slightest discomfort in wearing it. Thinks that the occasional changing of the jacket follows up the gradual correcting of the deformity, and in this way not only would the *cure* be effected, but that the *deformity* in many of these cases would also be remedied, which would not be done so well by wearing the *first* one put on. The little patient was jolly and happy, and had good active use of himself and seemed to enjoy the comfort afforded by his corset.

DRS. WILLIAMS and MCHATTON also exhibited patients with badly injured hands, the results illustrating the principle of conservative surgery done under the antiseptic *régime.* The cases were both railroad injuries, producing compound comminuted fractures of the bones of the hand and fingers; both saved useful hands with the loss of only one finger in one and a thumb in the other. In the latter case an attempt had been made to save the thumb, but gangrene supervened, and the amputation was done just at the margin of demarcation; a very good flap was secured, and union by first intention, without a drop of pus was the result, and in less than a month patient was dismissed entirely well.

DR. H. J. WILLIAMS, the essayist of the evening, now read a valuable and exhaustive paper on "Amputation and Dressings." (See page 268.)

DR. MCHATTON, in discussing Dr. Williams's paper said that Dr. Williams had so thoroughly covered the subject as to leave but little to be said; he agreed with Dr. Williams *in toto.* He mentioned one point which he had not seen mentioned in any of the books, or heard emphasized by any of the teachers or surgeons, viz., the influence of the weather, and the time of day for operations. We all know the bad feeling and general malaise incident to an east wind and bad weather. We alse know that the early morning brings with it the freshness and vigor of the pure morning air, and, other things being equal, he would defer an operation until a bright day and clear weather, and would always, where it could be done, prefer to operate in the morning. His recorded cases of railroad surgery consisted of twenty major and forty minor operations, done under strictly antiseptic rules, with absolutely perfect and satisfactory results.

DR. SHORTER, who had been appointed to discuss Dr. Williams's paper, said that he had been greatly entertained in listening to Dr. Williams's paper. The doctor's paper had dealt with amputations especially of the limbs, and he thought the same general principles would obtain in amputations of other members—the tongue, penis, etc. Mentions a caution which should always be used in the use of Esmarch's bandage, viz., that of too much pressure and the consequent injury to the nerves under the bandage, especially so with the round elastic bandage. The doctor's special line of work allows little opportunity for witnessing or participating in general surgery.

DR. FERGUSON takes issue with Dr. Williams as to the manner of making flaps in amputations of the limbs; he prefers the oblique method rather than the *circular* amputations. Thinks the flaps can be more evenly coaptated, the two angles of the wound will not protrude out as in the circular incision, and the stump will be left in a more uniform, well-rounded condition, and in much better shape for an artificial limb.

DR. WILLIAMS, in reply to Dr. Shorter, said that he never used the round elastic Esmarchs bandage, and now never rolled the limb from the extremity to point above amputation, as was formerly recommended. Two years ago heard a paper read by Dr. Senn before the National Association of Railroad Surgeons, and learned from him that to elevate the limb for five to ten minutes and apply bandage just above point of amputation was better than the old method; then using a wide bandage and using due caution against improper constriction of the soft tissues. In reply to Drs. Ferguson and Taylor, prefers the circular incision for the reason that the superabundance of tissue on the posterior part of the leg, for instance, would throw the cicatrix on the anterior face of the stump, and would leave a better rounded stump of healthy cushion for an artificial limb. Reference should always be had in all amputations, especially of the lower limbs, to the future use of an artificial limb.

K. P. MOORE, M.D., Reporter.

---

### GARGLE FOR SIMPLE TONSILLITIS.

In *Revista Clinica Thrapeutica*, the following formula is recommended in simple tonsillitis:

℞ Borax.................................................. 6 gms. (ʒ jss).

Tinct. of benzoin..........................................15 gms. (ʒ iv).

Rose water..........................................ad 130 gms. (℥ vss).

S. P. Gargle frequently with this mixture.

—*Medical and Surgical Reporter.*

